# Editorial: Stress and Cognition

**DOI:** 10.3389/fpsyg.2017.00970

**Published:** 2017-06-09

**Authors:** Jianhui Wu, Jin Yan

**Affiliations:** ^1^Institute of Affective and Social Neuroscience, Shenzhen UniversityShenzhen, China; ^2^School of Psychology and Mental Health, Second Military Medical UniversityShanghai, China

**Keywords:** human, stress, cognition, brain, individual difference

Animal research over many decades has established that stress and stress hormones have a profound influence on the brain, primarily on the hippocampus, amygdala, and prefrontal cortex (PFC) (Chattarji et al., 2015). This topic in human populations has increasingly been the focus of more research over the past 20 years (Qin et al., 2009; Schwabe and Wolf, 2010; Weymar et al., 2013; Wu et al., 2014). In this Research Topic of Frontiers in Psychology, a host of new empirical findings on the relationship between stress and cognition in humans are presented via studies employing multidisciplinary methods, including behavioral testing, event-related potentials (ERPs), and functional magnetic resonance imaging (fMRI).

Behavioral results in this research topic showed the detrimental effects of acute stress on executive functions (Starcke et al.), hand–eye coordination, mental rotation, and spatial memory (Hou et al.), and an impulsive responsiveness for an arithmetic task under acute stress (i.e., faster RTs and less accurate responses) (Qi et al.); whereas risk perception about imagined risky situations increased under acute stress (Sobkow et al.). On the brain level, the effect of acute stress is reflected in decreased amplitude of the P2 ERP component (most likely reflecting attentional resource allocation) time-locked to the onset of multiplication formulas during an arithmetic task (Qi et al.); and decreased neural efficiency during a well- trained perceptual task, i.e., more involvement of brain activation in primary and secondary somatosensory cortices is required to produce optimal behavioral performance (Bierzynska et al.).

The relationship between chronic stress and impaired cognition has also been well reported in the literature (Cerqueira et al., 2007; Liston et al., 2009; Wu et al., 2014). The case study in this Research Topic (Leung et al.) suggested that chronic perceived stress could be one of the modulating factors influencing neuroplasticity and behavioral benefits from cognitive training. Two participants with a history of stroke and a difficulty with working memory (WM) underwent a course of auditory WM training (n-back task) for 6 weeks with training conducted 5 days each week. Only the participant who had lower level of perceived chronic stress demonstrated improvements on n-back performance after training; while neural activation in the fronto-parietal regions critical for WM was decreased for this participant, suggesting increased neural efficiency of WM. There was no such effect, however, for the participant who had higher levels of perceived chronic stress. These results highlight the need to evaluate perceived chronic stress when studying neuroplastic changes in people with stroke. Nevertheless, this conclusion needs to be replicated in future studies with a larger sample size.

One of the main challenges of research on human stress is how to induce stress in the laboratory. Acute psychological stress is normally elicited by variable laboratory stress induction procedures in the papers included in this special topic as well as in the literature, such as frustration stress induced by a difficult tactile task with negative feedback (Bierzynska et al.), a mental arithmetic task (Sobkow et al.), time pressure when performing an arithmetic task (Qi et al.) and the Paced Auditory Serial Addition Test (Starcke et al.; Lejuez et al., 2003). This variability may lead to reduced comparability between results from different stressors. Different stressors may elicit unique stress response patterns and may produce unique effects on cognition. This suggests the need for standardized laboratory procedures when comparing results across studies in the field of human stress. For example, the TSST (Trier Social Stress Test) is a well-known and widely used procedure to induce social psychological stress (Kirschbaum et al., 1993; Buchanan et al., 2009). More standardized laboratory procedures including different nature of stressors are necessary as well.

The other issue related to stressors in human research, including studies in this Research Topic, is that only moderate level of stress may be induced in laboratory due to ethical limitations. The effects of moderate stress, however, may not have the same effects on cognition as higher levels of stress, such as those in animal researches or in real-world stressors. Animal studies have suggested an inverted-U, but not a linear, relationship between stress and behavior (Sapolsky, 2015). The other choice is to use naturally occurring life events, such as trauma exposure. Longitudinal follow-up studies of individuals before and after trauma may be beneficial for understanding the relationship of stress with cognitive and health outcomes. However, collecting data from these trauma-exposed individuals presents a big challenge for researchers. Future work should target likely trauma victims such as soldiers and other high-risk populations, in order to address these issues longitudinally.

The collection of articles in this Research Topic reflects the continued research of interest on the relationship between stress and cognition in humans, in spite of methodological challenges. Findings from these studies in humans replicated conclusions from animal models, including decreased activation in the PFC and hippocampus, but amygdala hyper-responsivity under stress, and are in line with a general assumption of a non-rational behavior and reflexive (not reflective) brain state under stress (Arnsten, 2015).

## Author contributions

JW wrote this manuscript. JY revised this manuscript.

### Conflict of interest statement

The authors declare that the research was conducted in the absence of any commercial or financial relationships that could be construed as a potential conflict of interest.
